# Synthesis, *in-vitro* inhibition of cyclooxygenases and *in silico* studies of new isoxazole derivatives

**DOI:** 10.3389/fchem.2023.1222047

**Published:** 2023-09-06

**Authors:** Waqas Alam, Haroon Khan, Muhammad Saeed Jan, Umer Rashid, Ali Abusharha, Maria Daglia

**Affiliations:** ^1^ Department of Pharmacy, Abdul Wali Khan University Mardan, Mardan, Pakistan; ^2^ Department of Pharmacy, Bacha Khan University, Charsadda, Pakistan; ^3^ Department of Chemistry, COMSATS University Islamabad-Abbottabad Campus, Abbottabad, Pakistan; ^4^ Optometry Department, Applied Medical Sciences College, King Saud University, Riyadh, Saudi Arabia; ^5^ Department of Pharmacy, University of Naples Federico II, Naples, Italy; ^6^ International Research Centre for Food Nutrition and Safety, Jiangsu University, Zhenjiang, China

**Keywords:** synthesis, new isoxazole derivatives, pharmacokinetics, physicochemical properties, SwissADME profile, drug likeness

## Abstract

Isoxazole belongs to the class of five-membered heterocyclic compounds. The process of developing new drugs has significantly gained attention due to inadequate pharmacokinetic and safety attributes of the available drugs. This study aimed to design a new diverse array of ten novel isoxazole derivatives via Claisen Schmidt condensation reaction. *In vitro* COX-1/2 anti-inflammatory assay, *in silico* molecular docking of potent compounds, Molecular docking simulation, and SwissADME pharmacokinetic profile were investigated in this research. The *in vitro* COX-1 and COX-2 enzyme inhibitory assay showed that almost all the tested compounds exhibited anti-inflammatory effects whereas **C6**, **C5**, and **C3** were found to be the most potent COX-2 enzyme inhibitors among the tested compounds and are good candidates for selective COX-2 inhibitors. *In silico* molecular docking studies coupled with molecular dynamic simulation has been done to rationalize the time-evolved mode of interaction of selected inhibitor inside the active pockets of target COX-2. The binding orientations and binding energy results also showed the selectivity of compounds towards COX-2. Physicochemical properties, pharmacokinetic profile, lipophilicity, water solubility, drug metabolism, drug-likeness properties, and medicinal chemistry of the synthesized isoxazole derivatives were assessed. The SwissADME (absorption, distribution, metabolism, and excretion) database was used to assess the physicochemical properties and drug-likeness properties of the synthesized isoxazole derivatives. All the compounds were shown high GI absorption except Compound 7 (**C7**). Compound 1 (**C1**) and Compound 2 (**C2**) were found to cross the blood-brain barrier (BBB). Lipinski’s rule of five is not violated by any of the ten synthesized isoxazole derivatives. It was predicted with the SwissADME database that **C2**, **C5**, **C6**, **C7**, and **C8** are potent inhibitors of cytochrome (CYP) subtype CYP-2C19. A subtype of CYP-2C9 was inhibited by **C4** and **C7**. The medicinal chemistry of all the compounds **C1**-**C10** showed no PAIN (Pan assay interference compounds) alerts. The improved gastrointestinal (GI) absorption and BBB permeability of **C1** and **C2** can provide a future prospective for new researchers in the medicinal field to investigate the compounds for the management of chronic diseases. The synthesized isoxazole compounds showed excellent *in vitro* COX-1/2 enzymes anti-inflammatory investigations, *in silico* studies, good physicochemical properties, and improved pharmacokinetic profile which will be further investigated via *in vivo* anti-inflammatory activities. Moreover, to further support our findings of the computational research and *in vitro* studies, an *in-vivo* pharmacokinetic profile is suggested in the future.

## 1 Introduction

Heterocyclic compounds have gained attention as they serve as a link between chemical and biomedical research. These compounds are the subject of extensive modern research worldwide (Karthikeyan et al., 2009; Banoglu et al., 2016). Isoxazole belongs to the class of five-membered heterocyclic compounds. An oxygen atom and a nitrogen atom occupy the adjacent positions of the two heteroatoms in the compound isoxazole. Two double bonds between carbon atoms give the molecule its unsaturated property. Isoxazole’s structural characteristics enable numerous non-covalent interactions, including hydrophilic interactions (overall hydrophilic profile with CLogP = 0.112), pi-pi stacks (unsaturated five-membered rings), and hydrogen bonds (hydrogen bond acceptor N and O). A common characteristic of organic compounds synthesized in the past few decades is that many of them may have contained a heterocycle ring (Ali et al., 2015; Shiro et al., 2015; Martorana et al., 2016; Zhu et al., 2018). Isoxazole and its derivatives serve as the basis for all organic synthesis structures. In addition, a variety of isoxazole derivatives, including natural ones like chalcones and hispolons, have an anti-inflammatory effect, immunomodulatory, anticancer, antimicrobial, anticonvulsant, anti-tubercular, anti-diabetic, and antiviral effects (Vitale and Scilimati, 2017; Zimecki et al., 2018; Balaji et al., 2019; Vidhya et al., 2019; Shaik et al., 2020). Researchers have reported that isoxazoles have analgesic and anti-inflammatory effects, and their chemistry has long been a fascinating area of study (Rajanarendar et al., 2015; Arya et al., 2021).

A review of the literature found that many substituted isoxazole had been synthesized using a variety of synthetic methods. In 1903, Claisen made the first discovery of the chemistry of isoxazole when he created the first compound in the family, isoxazole, by oximatingpropargylaldehydeacetal (Claisen, 1903). According to a review of the literature, changing different groups on the isoxazole ring results in varied activities. Many medications with isoxazole nuclei that are currently on the market fall into various therapeutic activity categories, leading to the development of numerous methods for the synthesis of this important component (Agrawal and Mishra, 2018). Celecoxib, etoricoxib, valdecoxib, and rofecoxib are members of the Coxibs family (Isoxazole derivatives), which are regarded as great achievements in this field because they demonstrated significant selectivity towards cyclooxygenase-2 (COX-2). However, valdecoxib and rofecoxib still have some cardiac side effects and have been taken off the market (Jackson and Hawkey, 2000; Micklewright et al., 2003). Its consumption is typically also accompanied by gastrointestinal (GI) adverse effects, which is a significant medical and economic issue (Abdel-Tawab et al., 2009). Hepatotoxicity, cardiovascular effects, platelet malfunction, and GI bleeding are the adverse effects that are linked to long-term consumption of Coxibs (Bergh and Budsberg, 2005; Chikkula and Sundararajan, 2017).

Coxibs have the same diaryl structure with a core ring composed of five members, such as pyrazole, pyridine, oxazole, or oxole, and the previously developed isoxazole, which has shown a positive anti-inflammatory action and a potential specificity for COX-2 (Abdelall et al., 2017). Specifically, the volume of COX-2 is greater than that of COX-1 because of a side pocket that can hold a big aryl. Hence, the yield of produced compounds would rise with the use of preferred isoxazole, aryl substituted furoxan, or fused isoxazole. To treat these GI issues, certain aryls, such as the well-known furoxan, generate nitroxyl (HNO), a physiological version of nitric oxide (NO) (Cena et al., 2003; Miller and Megson, 2007; de Carvalho et al., 2010). Moreover, the isoxazole might preserve COX-2 selectivity while having fewer adverse effects due to its specialized anti-inflammatory pharmacophore properties (Micklewright et al., 2003).

Researchers have been paying close attention to isoxazole derivatives because of their intriguing biological and pharmacological effects (Hamama et al., 2017). The SwissADME investigations are expected to minimize the likelihood of late-stage dropout in drug discovery, and improve screening and testing of the most potential compounds (Snoussi et al., 2023). The current research aims to develop new series of isoxazole derivatives, assess their *in vitro* COX/1-2 anti-inflammatory assays, investigate the *in silico* molecular docking and molecular docking simulation of the potent compounds, and provide new isoxazole derivatives with better pharmacokinetic profile and lesser side effects.

## 2 Materials and methods

### 2.1 Chemistry

#### 2.1.1 Synthesis of 5-acetyl-3,4-dihydropyridine-2-one derivatives

In the first step, 5-acetyl-3,4-dihydropyridine-2-one derivatives were synthesized by using Biginelli reaction condition. Tin (II) chloride dihydrate was used as a Lewis acid catalyst and Reflux conditions were used to carry out the reactions in acetonitrile (MeCN).

#### 2.1.2 Synthesis of chalcone derivatives

In the second step, the synthesized 5-acetyl-3,4-dihydropyridine-2-one derivatives were subjected to Claisen Schmidt condensation reaction with substituted aldehydes to form chalcone derivatives. The reactions were carried out in 40% alcoholic potassium hydroxide solution.

#### 2.1.3 Synthesis of Isoxazole derivatives

**SCHEME 1:**
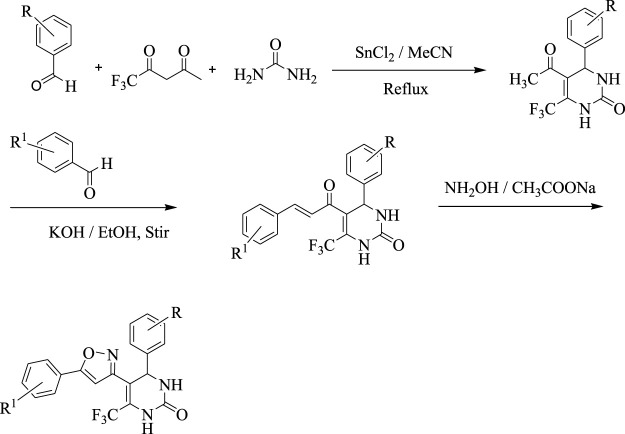
Synthesis of isoxazole derivatives

A chalcone (0.015 mol), sodium acetate (0.015 mol), and hydroxylamine hydrochloride (1.04 g, 0.015 mol) were combined and refluxed in ethanol for 7–8 h. TLC was used to keep track of when a reaction had finished. In order to concentrate the mixture, the solvent will be removed under reduced pressure. The mixture will then be placed into ice water, filtered, dried, and recovered from ethanol (Scheme 1).

##### 2.1.3.1 5-(5-methylisoxazol-3-yl)-4-phenyl-6-(trifluoromethyl)-3,4-dihydropyrimidin-2(1H)-one (C1)

^1^H NMR (400 MHz, DMSO-*d*_*6*_) δ 9.95 (br s, 1H, NH), 7.76 (s, 1H, NH), 7.24–7.20 (m, 5H, ArH), 5.14 (s, 1H, *J* = 3.32 Hz, 1H), 6.19 (s, 1H, C=CH), 2.38 (s, 3H, CH_3_).^13^C NMR (100 MHz, DMSO-*d*_*6*_) δ 164.7, 158.1, 152.9, 141.3, 132.2, 129.0, 128.4, 127.5, 125.1, 122.9, 104.9, 104.8, 104.8, 50.1, 12.1.

##### 2.1.3.2 4-(4-methoxyphenyl)-5-(5-methylisoxazol-3-yl)-6-(trifluoromethyl)-3,4-dihydro- pyrimidin-2(1H)-one (C2)

^1^H NMR (400 MHz, DMSO-*d*_*6*_) δ 9.92 (br s, 1H, NH), 7.76 (s, 1H, NH), 7.31 (d, 2H, *J* = 8.4 Hz,ArH), 6.94 (d, 2H, *J* = 8.4 Hz,ArH), 5.15 (s, 1H, *J* = 3.32 Hz, 1H), 6.18 (s, 1H, C=CH), 3.82 (s, 3H, OCH_3_), 2.38 (s, 3H, CH_3_).^13^C NMR (100 MHz, DMSO-*d*_*6*_) δ 164.6, 158.9, 156.6, 152.9, 152.9, 136.4, 132.2, 127.9, 124.8, 122.6, 114.24, 104.8, 104.2, 55.3, 50.1, 12.2.

##### 2.1.3.3 4-(3-hydroxy-4-methoxyphenyl)-5-(5-phenylisoxazol-3-yl)-6-(trifluoromethyl)-3,4-dihydropyrimidin-2(1H)-one (C3)

^1^H NMR (400 MHz, DMSO-*d*_*6*_) δ 9.94 (br s, 1H, NH), 7.75 (s, 1H, NH), 7.63 (br s, 1H, OH), 7.56–7.51 (m, 5H, ArH), 6.94–6.90 (m, 2H, ArH), 6.81 (s, 1H, ArH), 5.13 (s, 1H, *J* = 3.36 Hz, 1H), 6.17 (s, 1H, C=CH), 3.80 (s, 3H, OCH_3_).^13^C NMR (100 MHz, DMSO-*d*_*6*_) δ 159.8, 153.1, 150.2, 147.4, 146.5, 134.5, 132.4, 132.1, 130.8, 128.8, 127.4, 124.5, 122.4, 118.7, 114.1, 112.4, 105.4, 105.4, 101.9, 56.2, 50.1.

##### 2.1.3.4 5-(5-([1,1′-biphenyl]-4-yl)isoxazol-3-yl)-4-(4-methoxyphenyl)-6-(trifluoromethyl)-3,4-dihydropyrimidin-2(1H)-one (C4)

^1^H NMR (400 MHz, DMSO-*d*_*6*_) δ 9.93 (br s, 1H, NH), 7.98 (d, 2H, *J* = 8.8 Hz,ArH),7.76 (s, 1H, NH), 7.67 (d, 2H, *J* = 8.8 Hz,ArH), 7.51–7.47 (m, 5H, ArH), 6.18 (s, 1H, C=CH), 5.13 (s, 1H, *J* = 3.32 Hz, 1H), 3.84 (s, 3H, OCH_3_). ^13^C NMR (100 MHz, DMSO-*d*_*6*_) δ 159.7, 158.9, 153.6, 153.5, 150.1, 150.0, 141.9, 140.3, 136.3, 132.5, 132.2, 128.9, 128.2, 127.9, 127.9, 127.5, 127.2, 126.8, 124.5, 122.3, 114.2, 105.6, 105.5, 102.0, 55.3, 50.0, 50.0.

##### 2.1.3.5 4-(furan-2-yl)-5-(5-(furan-2-yl)isoxazol-3-yl)-6-(trifluoromethyl)-3,4-dihydropyrimidin-2(1H)-one (C5)

^1^H NMR (400 MHz, DMSO-*d*_*6*_) δ 9.92 (br s, 1H, NH), 7.76 (s, 1H, NH), 7.71–7.69 (m, 1H, CH), 7.55 (m, 1H, ArH), 6.89 (m, 1H, ArH), 6.70 (m, 1H, ArH), 6.30 (m, 1H, ArH), 6.17 (s, 1H, C=CH), 6.10–6.08 (m, 1H, ArH), 5.16 (s, 1H, *J* = 3.0 Hz, 1H).^13^C NMR (100 MHz, DMSO-*d*_*6*_) δ 158.2, 154.9, 153.7, 147.9, 143.6, 142.9, 141.8, 132.2, 124.6, 122.5, 112.8, 110.6, 109.1, 104.7, 103.4, 47.7.

##### 2.1.3.6 5-(5-(furan-2-yl)isoxazol-3-yl)-4-(4-methoxyphenyl)-6-(trifluoromethyl)-3,4-dihydro- pyrimidin-2(1H)-one (C6)

^1^H NMR (400 MHz, DMSO-*d*_*6*_) δ 9.92 (br s, 1H, NH), 7.96 (d, 2H, *J* = 8.8 Hz,ArH),7.74 (s, 1H, NH), 7.67 (d, 2H, *J* = 8.8 Hz,ArH), 7.54–7.51 (m, 1H, ArH), 6.31 (m, 1H, ArH), 6.18 (s, 1H, C=CH), 6.10–6.07 (m, 1H, CH), 5.14 (s, 1H, *J* = 3.32 Hz, 1H), 3.84 (s, 3H, OCH_3_). ^13^C NMR (100 MHz, DMSO-*d*_*6*_) δ 158.4, 157.6, 152.8, 148.3, 143.5, 142.3, 136.6, 132.2, 127.9, 124.5, 122.4, 114.2, 112.9, 112.8, 105.0, 104.9, 104.0, 55.3, 50.0.

##### 2.1.3.7 5-(5-(furan-2-yl)isoxazol-3-yl)-6-(trifluoromethyl)-4-(4-(trifluoromethyl)phenyl)-3,4-dihydropyrimidin-2(1H)-one (C7)

^1^H NMR (400 MHz, DMSO-*d*_*6*_) δ 9.94 (br s, 1H, NH), 7.73 (s, 1H, NH), 7.69 (d, 2H, *J* = 8.84 Hz,ArH), 7.53–7.50 (m, 1H, ArH), 7.38 (d, 2H, *J* = 8.84 Hz,ArH), 6.30 (m, 1H, ArH), 6.18 (s, 1H, C=CH), 6.10–6.07 (m, 1H, CH), 5.15 (s, 1H, *J* = 3.32 Hz, 1H). ^13^C NMR (100 MHz, DMSO-*d*_*6*_) δ 157.7, 153.0, 148.5, 143.7, 142.4, 142.0, 132.2, 130.7, 127.7, 126.5, 124.5, 124.2, 122.4, 112.9, 112.8, 105.0, 104.9, 104.1, 50.2.

##### 2.1.3.8 5-(5-(furan-2-yl)isoxazol-3-yl)-4-p-tolyl-6-(trifluoromethyl)-3,4-dihydropyrimidin-2(1H)-one (C8)

^1^H NMR (400 MHz, DMSO-*d*_*6*_) δ 9.94 (br s, 1H, NH), 7.75 (s, 1H, NH), 7.53–7.51 (m, 1H, ArH), 7.11 (d, 2H, *J* = 8.0 Hz,ArH), 7.04 (d, 2H, *J* = 8.0 Hz,ArH), 6.31–6.28 (m, 1H, ArH), 6.17 (s, 1H, C=CH), 6.10–6.07 (m, 1H, CH), 5.12 (s, 1H, *J* = 2.8 Hz, 1H), 2.31 (s, 3H, CH_3_). ^13^C NMR (100 MHz, DMSO-*d*_*6*_) δ 158.4, 157.6, 152.8, 148.3, 143.5, 142.3, 136.6, 132.2, 127.9, 124.5, 122.4, 114.2, 112.9, 112.8, 105.0, 104.9, 104.0, 55.3, 50.0.

##### 2.1.3.9 4-phenyl-5-(5-(pyridin-4-yl)isoxazol-3-yl)-6-(trifluoromethyl)-3,4-dihydropyrimidin-2(1H)-one (C9)

^1^H NMR (400 MHz, DMSO-*d*_*6*_) δ 9.94 (br s, 1H, NH), 8.79 (d, 2H, *J* = 8.8 Hz,ArH), 7.89 (d, 2H, *J* = 8.8 Hz,ArH),7.77 (s, 1H, NH), 7.22–7.17 (m, 5H, ArH), 5.14 (s, 1H, *J* = 3.32 Hz, 1H), 6.18 (s, 1H, C=CH).^13^C NMR (100 MHz, DMSO-*d*_*6*_) δ 163.4, 158.65, 152.9, 152.9, 149.3, 141.6, 132.5, 132.3, 132.1, 129.1, 128.3, 127.5, 124.5, 122.4, 122.0, 105.5, 105.5, 103.3, 50.1.

##### 2.1.3.10 4-(Pyridin-4-yl)-5-(5-(pyridin-4-yl)isoxazol-3-yl)-6-(trifluoromethyl)-3,4 dihydropyrimidin-2(1H)-one (C10)

^1^H NMR (400 MHz, DMSO-*d*_*6*_) δ 9.94 (br s, 1H, NH), 8.78 (d, 2H, *J* = 8.8 Hz,ArH), 8.45 (d, 2H, *J* = 8.8 Hz,ArH), 7.87 (d, 2H, *J* = 8.8 Hz,ArH),7.77 (s, 1H, NH), 7.33 (d, 2H, *J* = 8.8 Hz,ArH), 5.15 (s, 1H, *J* = 3.32 Hz, 1H), 6.18 (s, 1H, C=CH).^13^C NMR (100 MHz, DMSO-*d*_*6*_) δ 158.6, 152.9, 152.91, 150.1, 150.0, 149.9, 149.3, 144.9, 132.5, 132.2, 124.5, 122.6, 122.4, 122.0, 105.6, 105.5, 103.3, 50.0.

### 2.2 *In vitro* anti-inflammatory activities

#### 2.2.1 COX-1/2 enzymes inhibitory assay

Following the earlier reported standard procedure, the *in vitro* inhibitory potentials of the created compounds have been assessed *versus* both COX-1/2 enzyme inhibition while keeping in mind the involvement of two significant cyclooxygenases in inflammation. In a nutshell, the test specimens were made in progressively higher concentrations. Furthermore, enzyme solutions for COX-1 from a sheep source (CAT: C0733-5000UN, Sigma-Aldrich) and COX-2 from a human recombinant source (CAT: C0858) were developed. COX-1 has a concentration of 0.7–0.8 g/L, whereas COX-2 has a concentration of 300 units/mL. Both arachidonic acid (CAT: 150384) and lineolic acid (CAT: 60–33–3) from Sigma-Aldrich have been used as substrates for the aforementioned enzymes (Burnett et al., 2007; Jan et al., 2020). The COX-2 enzyme solution was primed at 300 U/mL levels. 10 μL of the enzyme solution was kept on ice (4°C) for 5–6 min before 50 µL of the required co-factor solution was added. This solution contains 1 mM hematin in a 0.1 M Tris-buffer with pH 8.0, 0.24 mM tetramethyl-p-phenylenediamine dihydrochloride (TMPD), and 0.9 mM glutathione. Then, for the next 5–10 min, the enzyme solution (60 µL) and experiment samples (20 µL) of different strengths has been kept at the normal temperature. Similar to that, 20 µL of 30 mM arachidonic acid was utilized to start the procedure. The mixture’s temperature was held at 37°C for an additional 15 min. A UV-visible spectrophotometer was used to determine the absorbance at 570 nm after which the process was halted by adding HCl. Using the absorbance value per unit time, the percentage of COX-2 inhibition was computed. Celecoxib served as the standard control in the present study.

### 2.3 Molecular docking analysis

Molecular Operating Environment (MOE 2016) was used to conduct docking investigations. Three-dimensional crystal structures of COX-1/2 enzymes were retrieved from protein data bank (PDB). The accession codes for the downloaded enzymes were 1EQG (COX-1) and 1CX2 (COX-2). Enzyme synthesis was followed by the use of the re-dock approach to validate the docking protocol. Co-crystallized ligands were re-docked into the active sites of their respective enzymes. The docking protocol showing root-mean square deviation less than 2.0 Å was used for docking of the synthesized compounds. In order to prepare the enzyme, ligands, and perform docking, we used our earlier described techniques (Alqahtani et al., 2022; Mahmood et al., 2022). After docking studies, Discovery Studio Visualizer was used to perform the analysis.

### 2.4 Molecular docking simulation (MDS)

MDS has been performed by means of the Desmond simulation package LLC (Schrodinger release 2023) to investigate the behavior of compound 6 in complex with 1CX2, selected based on its lowest binding energy. The simulations were conducted in the NPT ensemble at 300 K as well as 1 bar pressure for a duration of 100 ns, with a relaxation time of 1 ps for compound 6. The OPLS force field parameters were utilized, and long-range electrostatic interactions were computed using the particle mesh Ewald method with a Coulomb interaction cutoff radius of 9.0 Å. The explicit description of water molecules employed the simple point charge model. For later examination, trajectories were recorded at 100 ps The Simulation Interaction Diagram tool included in the Desmond MD package was used to analyze the behavior as well as the interactions that occur between ligand and proteins. By tracking the root mean square fluctuation (RMSF), root mean square deviation (RMSD), and binding interaction of ligand and protein atom locations over time, the stability of the MD simulations was evaluated (Hollingsworth, 2018; Polo-Cuadrado et al., 2022).

### 2.5 Physicochemical properties, pharmacokinetic profile, drug likeness and medicinal

Drug-like chemical space refers to substances with pharmacokinetic properties that allow them to last through the conclusion of clinical trials (phase I) (Bade et al., 2010). The physicochemical properties of the compounds, including their lipophilic nature and water solubility, pharmacokinetics proficiency, drug resemblance, and pharmaceutical chemistry, were estimated using the SwissADME database. The oral bioavailability radar, often known as the BOILED-Egg graph, was created by analyzing physicochemical properties. The SwissADME database was used for generating this graph (Daina et al., 2017; Ahmad et al., 2021).

## 3 Results and Discussion

### 3.1 Synthesis

The current research aimed to synthesize new isoxazole derivatives. A multistep protocol was followed to synthesize new isoxazole derivatives. Started with the synthesis of Biginelli Dihydropyrimidine derivatives by using aromatic aldehydes, urea, and 1,1,1-trifluoropentane-2,4-dione in acetonitrile solvent and tin (II) chloride as Lewis acid. The next step was the synthesis of chalcones from DHPM derivatives. The synthesis of the aforementioned compounds was carried out using Claisan-Schimdt reaction conditions. Finally, isoxazole derivatives were synthesized using hydroxylaminehydrochloride and sodium acetate (0.015 mol) in ethanol (Scheme 1).

### 3.2 *In vitro* inhibition of cyclooxygenases (COX-1/COX-2)

The result findings of *in vitro* COX-1 and COX-2 enzyme inhibition assay of the created compounds (**C1-10**) are represented in Table 1. Our compounds showed selectivity towards COX-2 enzymes in contrast to COX-1. In the COX-2 findings, it was established that **C6** was comparably most potent, having IC_50_ value of 0.55 ± 0.03 µM. Similarly, the second highest activity was displayed by **C5** giving an IC_50_ value of 0.85 ± 0.04 µM. The third highest activity was shown by **C3** having IC_50_ value _of_ 0.93 ± 0.01 µM. The computed selectivity index (SI) for our synthesized compounds (C1-10) was 94.83, 70.68, 24.26, 8.08, 41.82, 61.73, 113.19, 115.43, 59.34, and 3.09. A relatively high SI value indicates that the compound might be an appropriate option, particularly in individuals with stomach ulcers. As shown by the IC50 values in Table 1, all of our tested drugs were effective cyclooxygenase-II inhibitors.

**TABLE 1:** *In vitro* COX-1 and COX-2 enzymes inhibiton assay results of synthesized isoxazole derivatives using celecoxib as standard.

Compounds	Structure	IC_50_ (µM) ± SEM ^a^		SI ^b^
		COX-1	COX-2	
**C1**	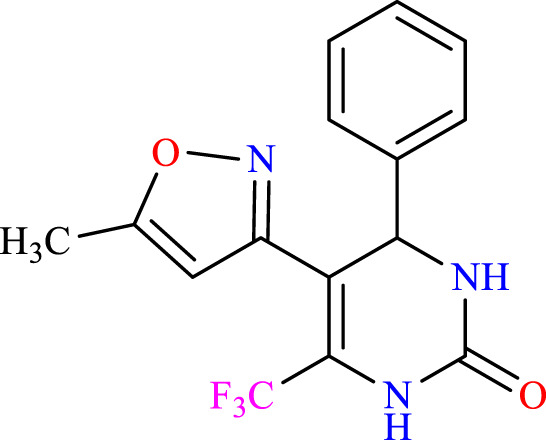	238.96 ± 2.30	2.52 ± 0.02	94.83
**C2**	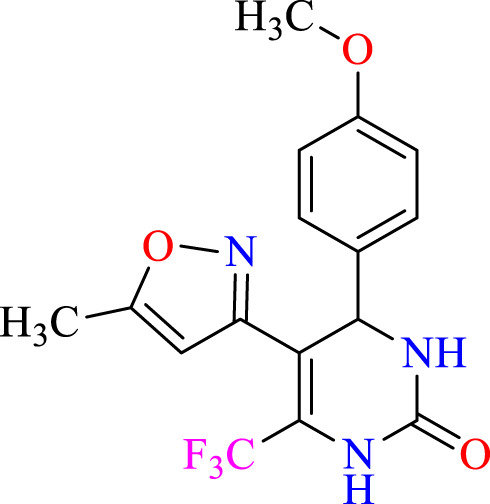	171.75 ± 1.90	2.43 ± 0.05	70.68
**C3**	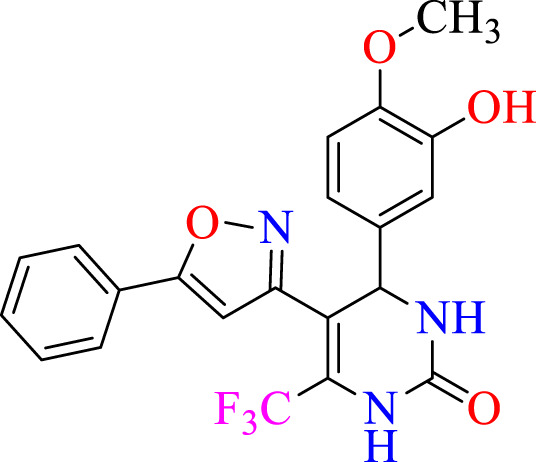	22.56 ± 0.90	0.93 ± 0.01	24.26
**C4**	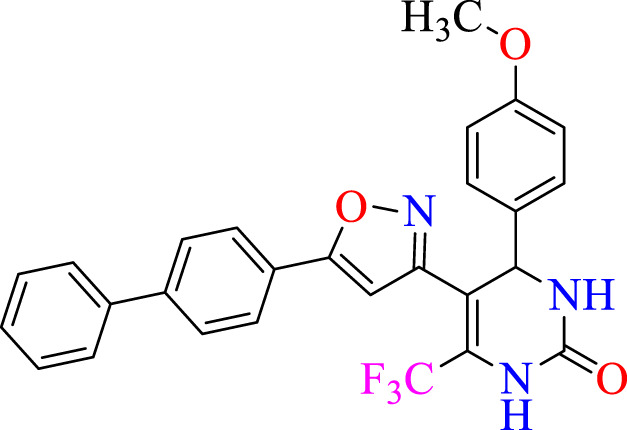	66.82 ± 1.84	8.27 ± 0.20	8.08
**C5**	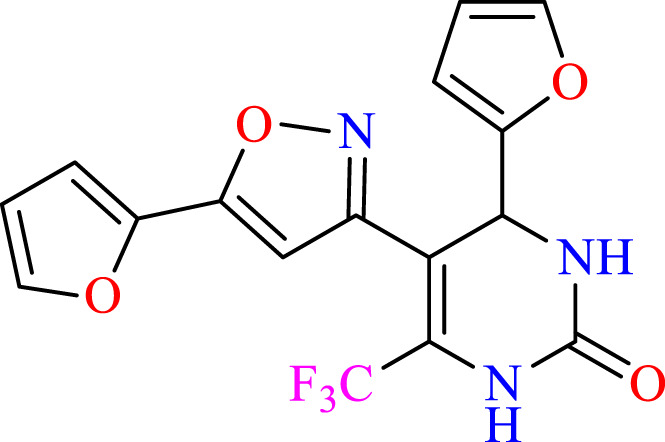	35.55 ± 1.49	0.85 ± 0.04	41.82
**C6**	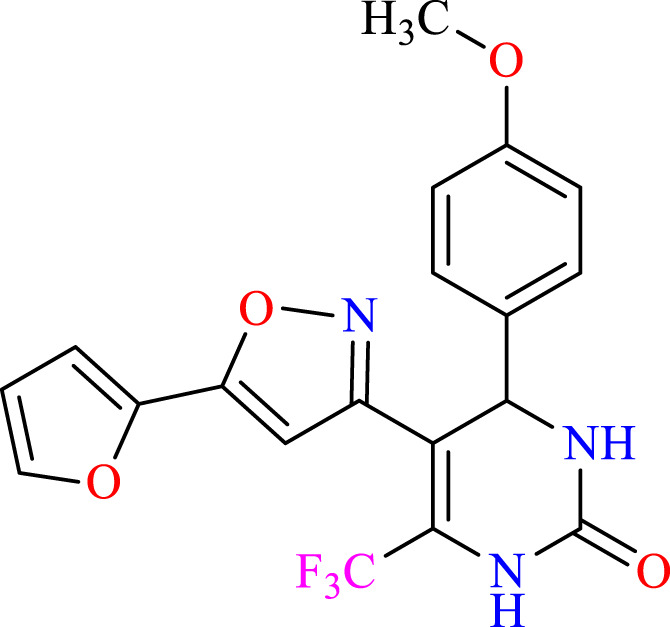	33.95 ± 1.31	0.55 ± 0.03	61.73
**C7**	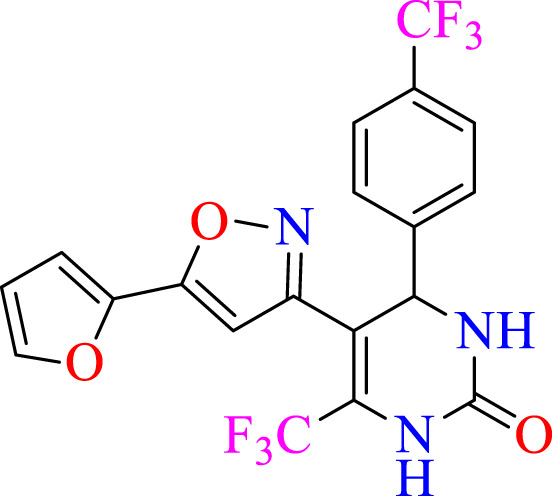	83.76 ± 0.96	0.74 ± 0.10	113.19
**C8**	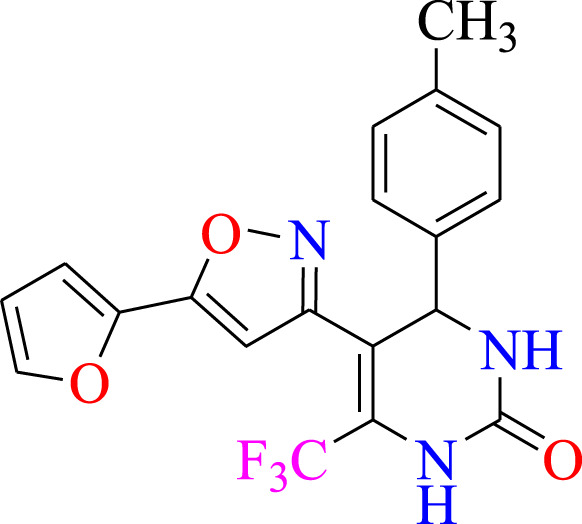	91.19 ± 0.51	0.79 ± 0.05	115.43
**C9**	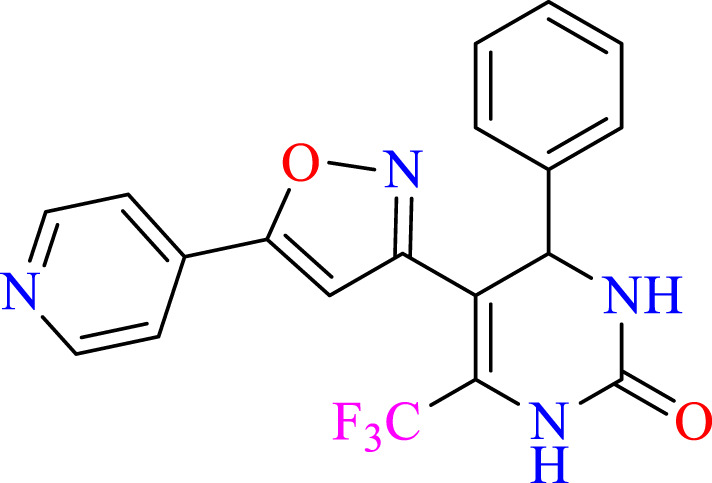	100.87 ± 1.17	1.70 ± 0.10	59.34
**C10**	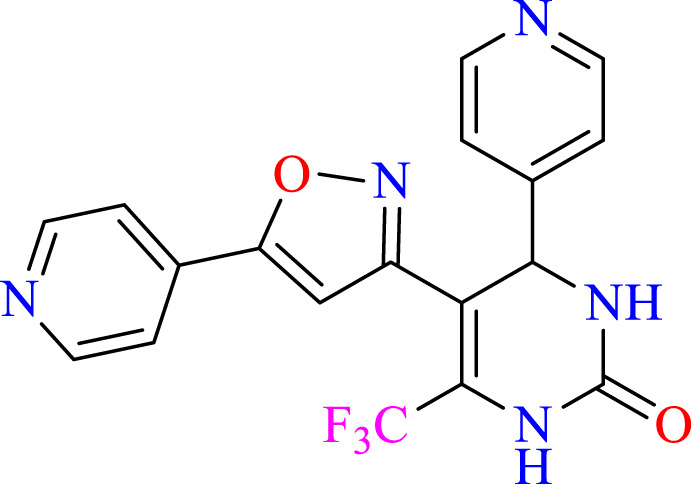	61.93 ± 1.11	20.03 ± 0.40	3.09
**Celecoxib**	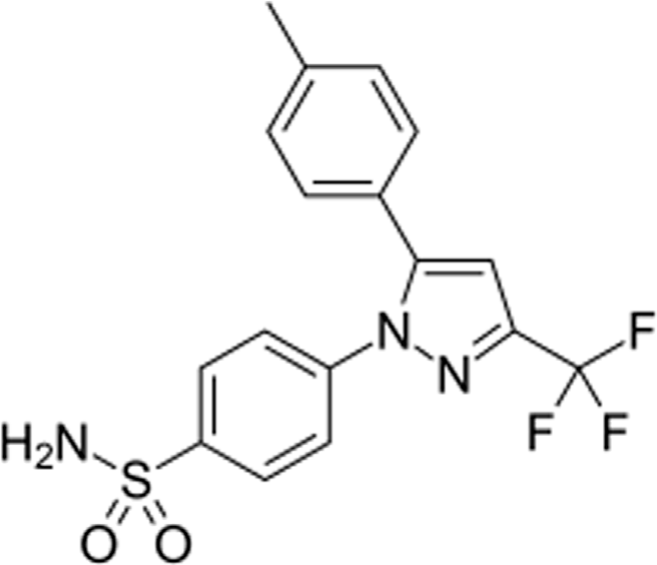	15.10 ± 0.90	0.05 ± 0.01	302

^a^ Data is expressed as mean ± SEM; *n* = 3.

^b^ Selectivity index: ratio of [IC50 (COX-2)]/IC50 (COX-1)].

### 3.3 Molecular docking analysis

Molecular Operating Environment (MOE 2016) was used to conduct docking investigations. Three-dimensional crystal structures of COX-1 and COX-2 enzymes were retrieved from protein data bank (PDB). The accession codes for the downloaded enzymes were 1EQG (COX-1) and 1CX2 (COX-2). Enzyme synthesis was followed by the use of the re-dock approach to validate the docking protocol. Co-crystallized ligands were re-docked into the active sites of their respective enzymes. The docking protocol showing root-mean square deviation less than 2.0 Å was used for docking of the synthesized compounds.

The analysis of binding orientations of compounds in the binding site of COX-2 displayed that the compounds exhibited hydrophobic and hydrophilic interactions with key amino acid residues present in the COX-2 specific pocket. These residues are; Val523, Phe518, Arg513, Ser353, Leu352 and His90. These strong interactions resulted in high and selective COX-2 potencies.

**FIGURE 1:**
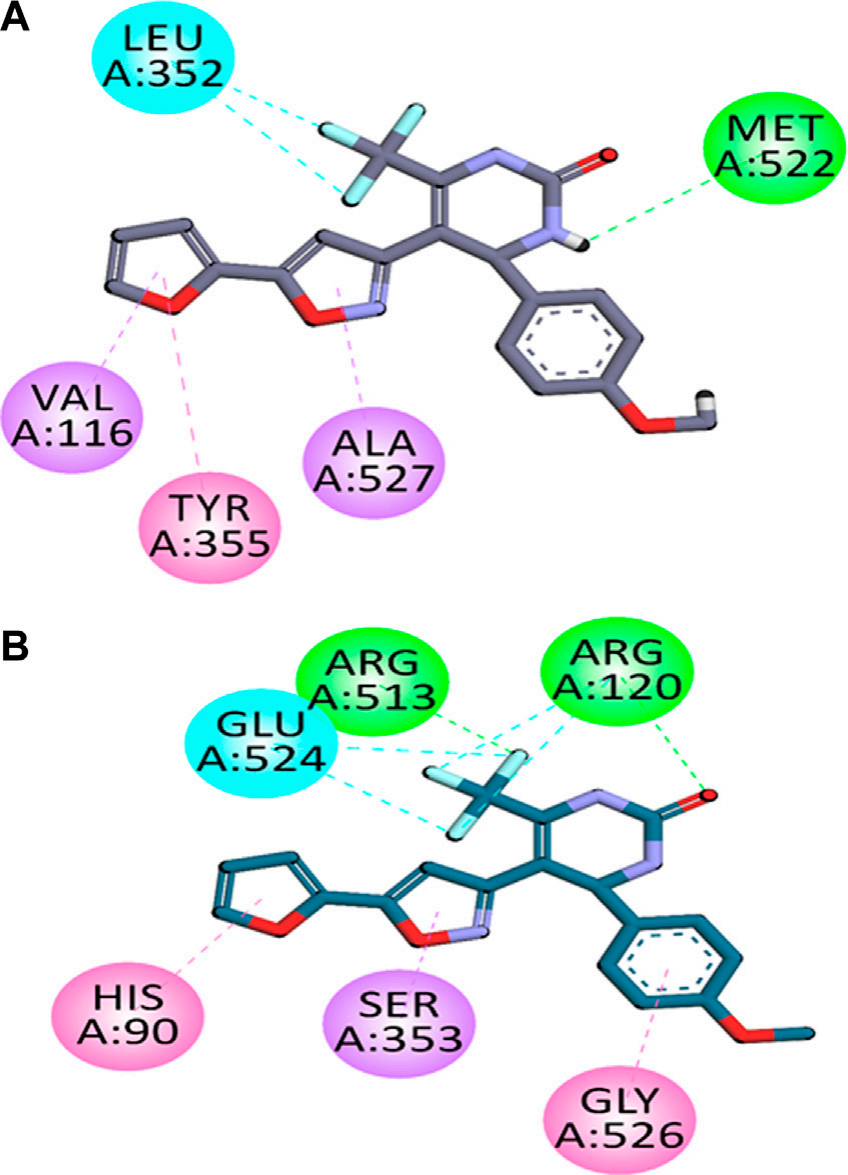
Compound C6’s two-dimensional interaction diagrams in COX-1 **(A)** and COX-2’s **(B)** binding positions.

The two-dimensional (2-D) interaction plots of the most potent compound **C6** at the binding sites of COX-1 as well as COX-2 are shown in Figure 1. The 2-D interaction plot of the most active compound **C6** in the COX-1 binding site is shown in Figure 1A. Fluorine atom forms halogen interactions with Leu352. It also establishes π-π stacking interactions with Tyr355 and π-σ interactions with Val116 and Ala127. The 2-D interaction plot of the most active compound **C6** in the COX-2 binding site is shown in Figure 1B. Fluorine atom forms hydrogen bond interactions with intensely positioned Arg513. It also establishes π-π stacking interactions with His90 and π-σ interactions with Ser353. The calculated binding energy value for **C6** in the binding position of COX-2 is −9.8854 kcal/mol. While for COX-1 it was −5.1812 kcal/mol. The binding energy results also showed the selectivity of compounds towards COX-2.

### 3.4 MD simulations

**FIGURE 2:**
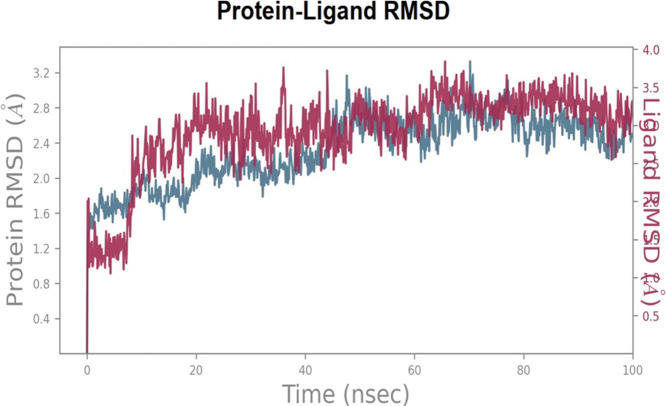
Root Mean Square Deviation RMSD trajectories of the COX-2-**C6** complex during the MD simulation, showing initial fluctuations (RMSD 2.0 A˚) until 10 ns followed by stable behavior (RMSD ∼1.0 A˚) for the remainder of the simulation.

In order to explore and analyze the enzyme-ligand interactions from an atomistic perspective and to assess the stability of the binding mode of C6 in complex with COX-2 enzyme during 100 ns of simulation, we performed molecular dynamics (MD) simulations. The major contacts and contributions over the molecular simulation trajectory of 100 ns are displayed in the Interaction Fraction Plots. The RMSD trajectories of the COX-2-**C6** complex during the molecular dynamics (MD) simulation were analyzed to understand the structural stability and fluctuations. Initially, from the start of the simulation until 10 ns, the complex exhibited slight deviations with an RMSD value of 2.0 A˚, indicating some degree of fluctuation during this early simulation time. However, after 10 ns and throughout the remainder of the simulation, **C6** demonstrated a more stable behavior, with the RMSD values maintaining an average deviation of around 1.0 A˚. This suggests that the compound reached a relative equilibrium state, showing minimal structural deviations from its initial conformation. The RMSD trajectories provide valuable insights into the dynamic behavior of the COX-2-**C6** complex, highlighting its stability and consistent conformational characteristics over time (see Figure 2 for visualization).

**FIGURE 3:**
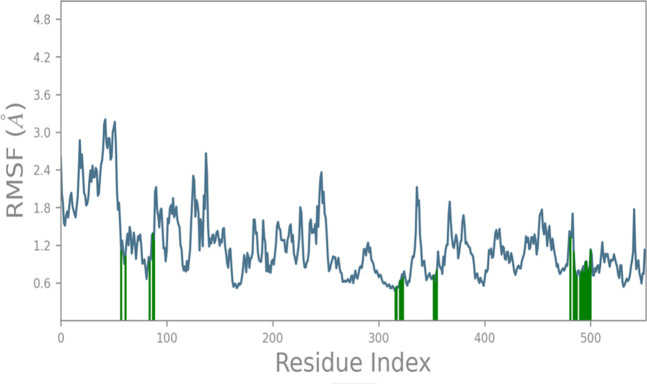
RMSF analysis of protein 1CX2 during 100 ns MD with **C6**, showing stable structure and notable fluctuations in N- & C-terminal of the catalytic domain (green bars indicate ligand-interacting residues).

The RMSF (root mean square fluctuation) analysis of protein 1CX2 during a 100 ns molecular dynamics (MD) simulation reveals local changes along the protein chain induced by compound 6. Residues of the protein that interact with the ligand are denoted by green-colored vertical bars. The RMSF analysis demonstrates a stable protein structure with an average deviation of approximately 1.45 Å throughout the simulation, providing a reliable foundation for further investigations. Notably, the RMSF profile highlights distinctive fluctuations in the N- and C-terminal regions of the catalytic domain, as observed in Figure 3, compared to other sections of the protein backbone amino acids.

**FIGURE 4:**
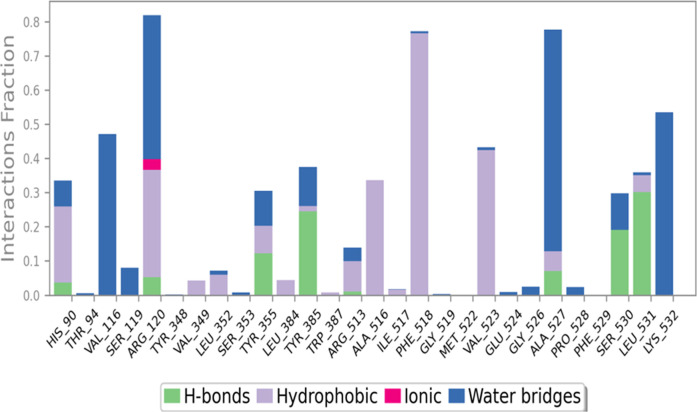
Interactions of **C6** with COX-2 enzyme during 100 ns MD simulation, depicted as stacked bar charts showing normalized interaction fractions (hydrophobic, H-bonds, ionic, and water bridges) over time.

Figure 4, provides a comprehensive depiction of the interactions between **C6** and the COX-2 enzyme throughout a 100 nanosecond molecular dynamics (MD) simulation. The figure consists of stacked bar charts that showcase the interaction fractions between the enzyme COX-2 and **C6**. These interactions are categorized based on their nature. Hydrophobic interactions are visualized in a purple color, hydrogen bonds in green, ionic interactions in fuchsia, and water bridges in blue. The stacked bar charts are normalized, ensuring that the values represented on the chart signify the percentage of time a specific interaction is sustained during the simulation. By examining this figure, one can gain insights into the relative prominence and duration of each interaction type, thereby aiding in the understanding of the molecular interactions between compound **C6** and the COX-2 enzyme.

### 3.5 Analyzing physicochemical parameters

The physicochemical parameters of the synthesized isoxazole derivatives (**C1**-**C10**) were evaluated, using the SwissADME database. Lipinski’s rule of five was not violated by any of the ten synthesized isoxazole derivatives. This indicates that their molecular structure makes them suitable for oral medications. Different physicochemical parameters, including topological polar surface area (TPSA), molecular weight (MW), the number of hydrogen bond donors (nHBD), No. of sp^3^ hybridized carbon out of total carbon count (F. Csp^3^), the number of hydrogen bond acceptors (NHBAs), molar refractivity (MR)and logP were computed and it was found that the synthesized isoxazole derivatives (**C1**-**C10**) were within the corresponding recognized limits of MW ≤ 500 g/mol, nHBD≤5, TPSA ≤140A^2^and LogP≤10 (Table 2). There were no rotatable bonds (nRB). TPSA values less than 140 A^2^ suggest that compounds had high permeability and bioavailability, which correspond to a drug’s capacity to diffuse into cells. The F. Csp3 calculated values for **C1**-**C10** were found to be in the range of 0.11–0.25. The nAHA values of **C1**-**C10** were measured in the range of 11–23. The synthesized isoxazole derivatives (**C1**-**C10**) have variations in the nRB and were in the range of 3–6. For the synthesized isoxazole derivatives **C1**-**C10**, the MR were found in varying degrees and were calculated, ranging from 82.57 to 134.97.

**TABLE 2:** The Physicochemical characteristics of Isoxazole derivatives were computed via the SwissADME database.

Ligands	Molecular formula	MW (g/mol)	nHA	nAHA	F. Csp3	nRB	nHBA	Nhbd	MR	TPSA (A^2^)
**C1**	C_15_H_12_F_3_N_3_O_2_	323.27	23	11	0.20	3	6	2	82.57	67.16
**C2**	C_16_H_14_F_3_N_3_O_3_	353.30	25	11	0.25	4	7	2	89.06	76.39
**C 3**	C_21_H_16_F_3_N_3_O_4_	431.36	31	17	0.14	5	8	3	111.55	96.62
**C4**	C_27_H_20_F_3_N_3_O_3_	491.46	36	23	0.11	6	7	2	134.97	76.39
**C5**	C_16_H_10_F_3_N_3_O_4_	365.26	26	15	0.12	4	8	2	87.57	93.44
**C6**	C_19_H_14_F_3_N_3_O_4_	405.33	29	16	0.16	5	8	2	101.80	89.53
**C7**	C_19_H_11_F_6_N_3_O_3_	443.30	31	16	0.16	5	10	2	100.31	80.30
**C8**	C_19_H_14_F_3_N_3_O_3_	389.33	28	16	0.16	4	7	2	100.27	80.30
**C9**	C_19_H_13_F_3_N_4_O_2_	386.33	28	17	0.11	4	7	2	100.83	80.05
**C10**	C_18_H_12_F_3_N_5_O_2_	387.32	28	17	0.11	4	8	2	98.63	92.94

nHA, No. heavy atom; MR, molar refractivity; M.W, molecular weight; F. Csp^3^, No. of sp^3^ hybridized carbon out of total carbon count; nAHA, No. arom. heavy atom; nHBA, No. H-bond acceptors; nRB, No. rotatable bonds; nHBD, No. H-bond donors; TPSA, topological polar surface area.

**FIGURE 5:**
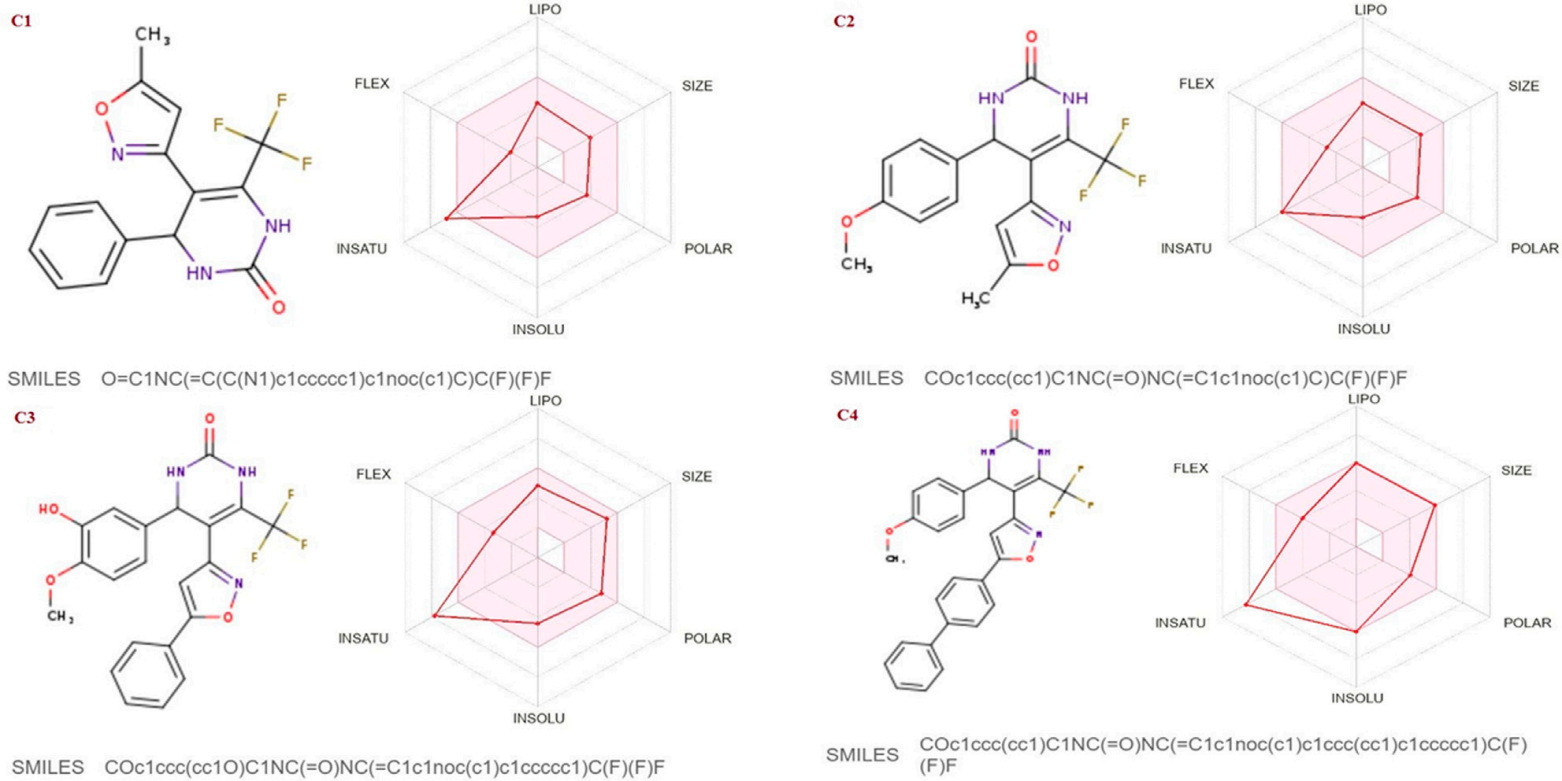
Lipophilicity and Oral bioavailability radar chart of C1-C4 synthesized Isoxazole derivatives predicted with Swiss ADME database.

**FIGURE 6:**
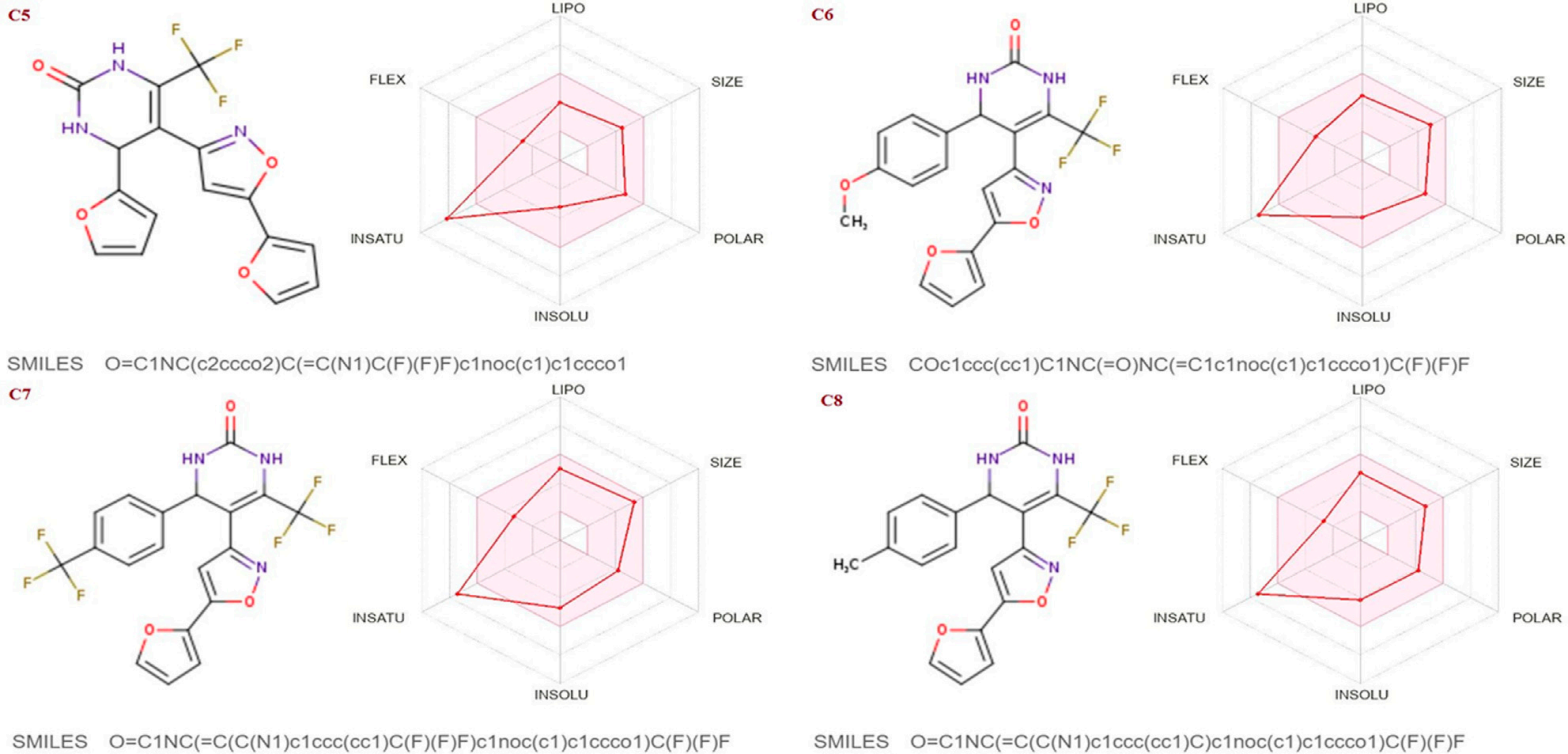
Lipophilicity and Oral bioavailability radar chart of C5-C8 synthesized Isoxazole derivatives predicted with Swiss ADME database.

**FIGURE 7:**
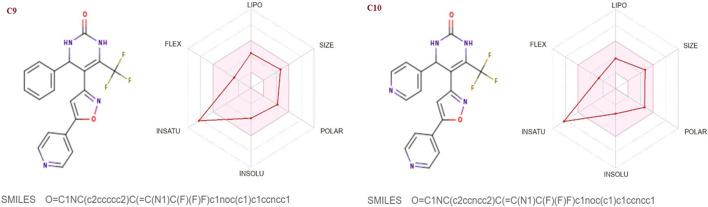
Lipophilicity and Oral bioavailability radar chart of C9-C10 synthesized Isoxazole derivatives computed with SwissADME database. The red line represents the oral drug bioavailability properties of the derivatives, while the pink zone represents the optimal physicochemical environment for oral drug bioavailability. FLEX, flexibility; LIPO, lipophilicity; INSATU, insaturation; INSOLU, insolubility; POLAR, polarity.

The SwissADME database was used to calculate the oral bioavailability and liphophilicity radar graph as shown in Figures 5–7. The 6 physicochemical properties of a molecule like insaturation, polarity, flexibility, insolubility, lipophilicity, and size are used in this graph. The optimum physicochemical atmosphere for oral medication bioavailability is shown by the pink zone, while the red line depicts the derivatives’ oral drug bioavailability qualities. All the synthetic isoxazole derivatives (**C1**-**C10**) fall within the range outlined by the pink zone in terms of their physicochemical nature (Figures 5–7). The instauration state is an outlier for all derivatives except **C2** (Figure 5).

### 3.6 Lipophilicity and water solubility

LogP_o/w_ is the log of the octanol/water partition coefficient, with a best-fit range of 0–3. Greater logP values indicate higher lipophilicity, which is determined by hydrogen bonding, molecular size, and polarity. All compounds’ log P_o/w_ (lipophilicity) values fell within the normal range [log P_o/w_< 5]. The log *p* values of synthesized (**C1**-**C10**) isoxazole derivatives were predicted in the range of 2.07–4.82 (Table 3) which reflects the partition of almost all synthesized derivatives into lipid compartments. Log S is defined as water solubility with a value of −4∼0.5 log mol/L. The log *p*-value corresponds to the log S value. The Log S results showed that **C1** and **C2** are moderately soluble in an aqueous state having log S values of −3.27 and −3.35 respectively. Whereas the Log S values of **C3**-**C10** predicted poor solubility except **C4**, which was found to be insoluble in the aqueous phase having Log S = −6.04. The lipid solubility and water solubility nature of **C1**-**C10** is summarized in Table 3.

**TABLE 3:** Lipophilicity and water solubility of Isoxazole derivatives computed by SwissADME database.

	Lipophilicity	Water solubility					
Ligands	Consensus Log P_o/w_	Log S (ESOL)	Solubility class	Log S (Ali)	Solubility class	Log S (SILICOS-IT)	Solubility class
**C1**	2.51	−3.27	Soluble	−3.06	Soluble	−5.85	Moderately soluble
**C2**	2.54	−3.35	Soluble	−3.22	Soluble	−5.96	Moderately soluble
**C3**	3.18	−4.42	Moderately soluble	−4.59	Moderately soluble	−7.45	Poorly soluble
**C4**	4.82	−6.04	Poorly soluble	−6.22	Poorly soluble	−10.48	Insoluble
**C5**	2.23	−3.20	Soluble	−3.05	Soluble	−6.38	Poorly soluble
**C6**	2.87	−3.91	Soluble	−3.87	Soluble	−7.26	Poorly soluble
**C7**	3.96	−4.70	Moderately soluble	−4.63	Moderately soluble	−7.98	Poorly soluble
**C8**	3.25	−4.15	Moderately soluble	−4.09	Moderately soluble	−7.53	Poorly soluble
**C9**	2.80	−3.81	Soluble	−3.53	Soluble	−7.56	Poorly soluble
**C10**	2.07	−3.14	Soluble	−2.69	Soluble	−7.19	Poorly soluble

### 3.7 Pharmacokinetics profile

**FIGURE 8:**
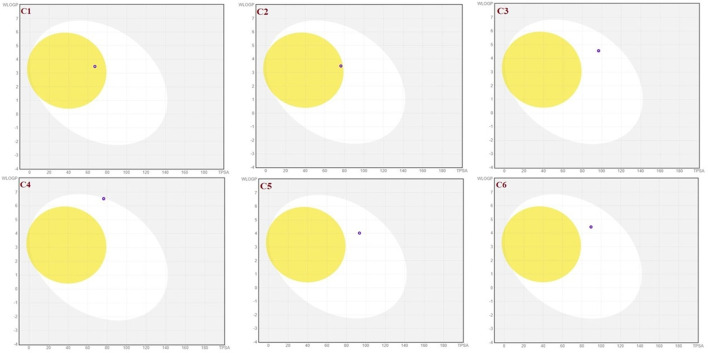
Boiled-egg graphical representation of compound C1 and C6 predicted via Swiss ADME data base.

**FIGURE 9:**
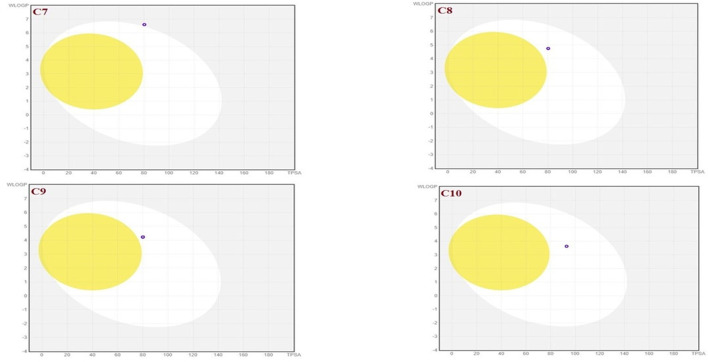
Boiled-egg graphical representation of compound C7 and C10 predicted by SwissADME data base.

To achieve optimal pharmacological outcomes, it is mandatory to understand a drug’s pharmacokinetics (PK). This suggests that each compound’s pharmacokinetic property may ultimately have an impact on a drug’s pharmacological action. The calculation of the SwissADME pharmacokinetic profile of synthesized Isoxazole derivative (**C1**-**C10**) has shown marked variation. Gastrointestinal (GI) absorption of all compounds (**C1**-**C10**) has shown high absorption except **C7** which showed low GI absorption. The bioavailability score was predicted for all the compounds to be 0.05. The SwissADME database predicted that all the compounds (**C1**-**C10**) do not cross the blood-brain barrier except **C1** and **C2** (Table 4). It was further concluded that all the synthesized isoxazole compounds (**C1**-**C10**) are substrates for *P*-glycoprotein. The boiled-egg graphical representation of **C1**-**C10** is analyzed by the SwissADME database and is shown in Figures 8, 9. Human intestine absorption is represented by the egg white region, central nervous system (CNS) penetration is represented by the egg yellow region, and additional routes besides the oral route are represented by the graph’s grey area. From the boiled egg graph, it was concluded that **C1** and **C2** have shown intestinal absorption and cross the BBB, whereas **C3**-**C10** have shown GI absorption and do not cross the BBB (Figures 8, 9).

**TABLE 4:** The Pharmacokinetic SwissADME Profile of Isoxazole derivatives.

Ligands	GI absorption	Bioavailability score	BBB permeant	P-gp substrate	CYP1A2 inhibitors	C YP2C19 inhibitors	CYP2C9 inhibitors	CYP2D6 inhibitors	CYP3A4 inhibitors
**C1**	High	0.55	Yes	Yes	No	No	No	No	No
**C2**	High	0.55	Yes	Yes	No	Yes	No	No	No
**C 3**	High	0.55	No	Yes	No	No	No	No	No
**C4**	High	0.55	No	Yes	No	No	Yes	No	No
**C5**	High	0.55	No	Yes	No	Yes	No	No	No
**C6**	High	0.55	No	Yes	No	Yes	No	No	No
**C7**	Low	0.55	No	Yes	No	Yes	Yes	No	No
**C8**	High	0.55	No	Yes	No	Yes	No	No	No
**C9**	High	0.55	No	Yes	No	No	No	No	No
**C10**	High	0.55	No	Yes	No	No	No	No	No

The drug metabolism of synthesized isoxazole derivatives (**C1**-**C10**) were analyzed via CYP-450 microsomal oxygenase system. It was predicted with the SwissADME database that **C2**, **C5**, **C6**, **C7**, and **C8** are potent inhibitors of subtype CYP-2C19 (Table 4). The subtype of CYP-2C9 was inhibited by **C4** and **C7**. The rest of the compounds did not show any effect on subtypes CYP-1A2, CYP-2D6, and CYP-3A4. The complete SwissADME pharmacokinetic profile of **C1**-**C10** is summarized in Table 4.

### 3.8 Drug likeness

Drug likeness of **C1** to **C10** was analyzed via the SwissADME database and it was found that all the derivatives did not show violation of Lipinski’s rule of five, Veber, Muegge, Egan, and Ghose rules of drug-likeness (Table 5). However, exceptions were also noted, such as **C4** violated Ghose rules (No; 3 violations: MW > 480, WLOGP>5.6, MR > 130) and Egan rules (No; 1 violation: WLOGP>5.88) while **C7** violated Gose rule (No; 1 violation: WLOGP>5.6) and Egan rules (No; 1 violation: WLOGP>5.88).

**TABLE 5:** Drug likeness and medicinal chemistry of Isoxazole derivatives calculated with SwissADME database.

Ligands	Drug Likeness rules					Medicinal chemistry			
	Lipinski	Ghose	Veber	Egan	Muegge	PAINS	Brenk	Lead likeness	Synthetic accessibility
**C1**	Yes; 0 violation	Yes	Yes	Yes	Yes	0 alert	0 alert	Yes	3.99
**C2**	Yes; 0 violation	Yes	Yes	Yes	Yes	0 alert	0 alert	No; 1 violation: MW > 350	4.06
**C 3**	Yes; 0 violation	Yes	Yes	Yes	Yes	0 alert	0 alert	No; 1 violation: MW > 350	4.38
**C4**	Yes; 0 violation	No; 3 violations: MW > 480, WLOGP>5.6, MR > 130	Yes	No; 1 violation: WLOGP>5.88	Yes	0 alert	0 alert	No; 2 violations: MW > 350, XLOGP3>3.5	4.74
**C5**	Yes; 0 violation	Yes	Yes	Yes	Yes	0 alert	0 alert	No; 1 violation: MW > 350	4.27
**C6**	Yes; 0 violation	Yes	Yes	Yes	Yes	0 alert	0 alert	No; 1 violation: MW > 350	4.30
**C7**	Yes; 0 violation	No; 1 violation: WLOGP>5.6	Yes	No; 1 violation: WLOGP>5.88	Yes	0 alert	0 alert	No; 1 violation: MW > 350	4.34
**C8**	Yes; 0 violation	Yes	Yes	Yes	Yes	0 alert	0 alert	No; 1 violation: MW > 350	4.33
**C9**	Yes; 0 violation	Yes	Yes	Yes	Yes	0 alert	0 alert	No; 1 violation: MW > 350	4.19
**C10**	Yes; 0 violation	Yes	Yes	Yes	Yes	0 alert	0 alert	No; 1 violation: MW > 350	4.24

### 3.9 Medicinal chemistry

The medicinal chemistry of the synthesized isoxazole derivative (**C1**-**C10**) was predicted by the SwissADME database and summarized in Table 5. All the isoxazole compounds **C1**-**C10** showed no PAIN (Pan Assay interference compounds) alerts viz devoid of frequent hitters, reactive substances, and α-screen artifacts (Table 5). No Brenk structural alert was detected in all the compounds (**C1**-**C10**). As the molecular weight of all compounds was greater than 350 (MW > 350) except **C1**, and thus **C2**-**C9** are not capable of lead likeness and caused violation. The lead likeness violation for **C2**, **C3**, **C5**, **C6**, **C7**, **C8**, **C9** and **C10** was found as No; 1 violation: MW > 350 whereas **C4** showed No; 2 violations: MW > 350, XLOGP3>3.5. The synthetic accessibility score for **C1**-**C10** was calculated in the range of 3.99–4.74 via Swiss ADME that reflects simple synthesis step reactions.

## 4 Discussion

Isoxazoles, as one of the vivid groups of aromatic heterocyclic compounds, were first discovered by Claisen in 1888, and have since been recognized as a special class of molecules in organic chemistry (Claisen and Lowman, 1888; Duc and Dung, 2021). The 5-membered heterocyclic molecule, isoxazole has oxygen and nitrogen atoms at positions 1 and 2, and its partially saturated analogues are known as isoxazolines, while its fully saturated analogue is known as isoxazolidin (Pandhurnekar et al., 2022). Isoxazoles, as one of the significant five-membered heteroaromatic ring structures, possess considerable pharmacological and biological activities in natural products, pharmaceutical agents, and preclinical candidates, comprising anticancer, antibacterial, antibiotic, and anti-inflammatory effects (Sun et al., 2015; Zhu et al., 2018; Pandhurnekar et al., 2022). As an example, valdecoxib has been proven to be a COX-2 inhibitor. Oxyacillin, a β-lactam antibiotic. Vernalis has been designed as an investigational anticancer candidate for the treatment of cancer (NVP-AUY922) (Severin et al., 2004; Jensen et al., 2008; Alajarín et al., 2011).

Isoxazoles and their derivatives have been recognized as significant and synthetically practical aromatic heterocycles. Particularly, polysubstituted isoxazole structures exhibit remarkable pharmacological and biological effects. As a result, in recent years, extensive investigations have focused on developing unique and effective synthetic techniques for the synthesis of structurally varied isoxazole scaffolds (Duc and Dung, 2021). Furthermore, the evaluation of medicinal ring systems reveals that the isoxazole ring is ranked 33rd out of 351 ring systems discovered in commercially available medications. Moreover, isoxazoles can be used as diverse synthetic intermediates in the highly effective synthesis of polyfunctionalized organic small molecules and functional materials. It is therefore more interesting to develop efficient and useful synthetic techniques for the rapid and easy assembly of structurally different polyfunctionalized isoxazoles (Taylor et al., 2014; Li et al., 2017; Vitale and Scilimati, 2017). In this study, Claisan-Schimdt reaction conditions were employed for the synthesis of these derivatives. Finally, isoxazole derivatives were synthesized (Scheme 1). The *in vitro* cyclooxygenase assay is commonly used for the assessment of the anti-inflammatory potential of the test compounds. Since an *in vitro* assay is a quick and simple method as compared to clinical research, scientists have suggested using *in vitro* assay to estimate the predicted levels of COX inhibition of the test compounds (Blain et al., 2002; Petrovic and Murray, 2010). In the present investigation, a COX-1 enzyme inhibition assay was performed to assess the *in vitro* anti-inflammatory potential of synthesized compounds. The IC_50_ (µM), or concentration at which 50% of an enzyme is inhibited, was used to assess the potency of the compounds. Our research findings established that almost all the synthesized tested compounds showed significant activity against *in vitro* COX-1 enzymes assay. Different concentrations of the tested compounds were assayed which showed excellent IC_50_ values. The tested compounds showed not excellent results against COX-1 enzyme. Among the tested compounds the significant IC_50_ value was observed for **C3**, **C5** and **C6** as compared to the standard drug.

The primary enzyme involved in inflammation is COX-2. Inflammatory mediators are produced by this inducible enzyme. Under normal settings, COX-2 is virtually completely absent from most tissues, but when there is inflammation or after exposure to mutagenic stimuli, its expression can be significantly elevated from 10 to 80 folds (DeWitt et al., 1993; Seibert et al., 1994). Prostaglandin (PG) and thromboxane (TX) production is inhibited by the majority of commercially available nonsteroidal anti-inflammatory medications (NSAIDs), which act by inhibiting both constitutive COX-1 and inducible COX-2. The therapeutic anti-inflammatory, analgesic, and antipyretic effects of NSAIDs are possibly due to COX-2 inhibition (Famaey, 1997). The potential of each synthesized compound to block the COX-2 enzyme was evaluated. The potency of the compounds was calculated as IC_50_ in µM, which is the amount of the compound that results in 50% inhibition of the enzyme. In our research investigations, we found out that all 10 newly synthesized isoxazole derivatives showed excellent dose-dependent COX-2 *in vitro* anti-inflammatory effects. All the compounds were found potent. Among the tested compounds, **C6**, **C5**, and **C3** showed significant IC_50_ values and established excellent COX-2 anti-inflammatory effects. The demonstrated IC_50_ values for all the tested compounds showed significant values comparable to the IC_50_ of standard drugs. In the current research, **C6** was observed significant and showed excellent COX-2 anti-inflammatory effect in a dose-dependent manner (0.55 ± 0.03 µM), having a minimum IC_50_ value as compared to the standard drug. Compound **C3** and **C5** also showed significant anti-inflammatory effects against COX-2 enzymes with minimum IC_50_ values of 0.93 ± 0.01 and 0.85 ± 0.04 µM respectively.

*In silico* molecular docking studies coupled with molecular dynamic simulations were also performed to rationalize the time-evolved mode of interaction of selected inhibitors inside the active pockets of target COX-2. The binding orientations and binding energy results also showed the selectivity of compounds towards COX-2. The two-dimensional (2-D) interaction plots of most compound **C6** in the binding sites of COX-1 and COX-2 are shown in Figure 1. The 2-D interaction plot of the most active compound C6 in the COX-1 binding site is shown in Figure 1A. Fluorine atom forms halogen interactions with Leu352. It also establishes π-π stacking interactions with Tyr355 and π-σ interactions with Val116 and Ala127. The 2-D interaction plot of the most active compound **C6** in the COX-2 binding site is shown in Figure 1B. Our *In silico* molecular docking studies confirmed that among the tested compounds **C6** was found most potent selective COX-2 inhibitor.

In the present research, the pharmacokinetic profile indicated that the synthesized isoxazole derivatives **C1**–**C10** followed Lipinski’s rule of five, a crucial indicator in drug discovery. Any medicinal compound that violates even one of the guidelines of Lipinski’s rule may have poor permeability or absorption (Pathak et al., 2017). The percentage of sp^3^ carbon atoms in the total carbon count is known as Fsp^3^. This describes the complexities of the molecule’s spatial structure and affects carbon saturation. Fsp^3^ can be optimized at a value of ≥0.42 because 84% of commercial medicines fulfill this criterion (Kombo et al., 2013). Conversely, a higher Fsp^3^ score is not a guarantee of improved performance and can make chemical synthesis more difficult, therefore sp3 level needs to be enhanced within a range (Gerlach et al., 2019). Natural compounds are an abundant source of medications since synthetic products typically possess a lesser proportion of sp3 than natural compounds (Jia et al., 2020). Our research findings showed that Fsp^3^ values are within range and thus are promising candidates for commercial grade with improved therapeutic performance and a convenient synthetic scheme.

With more than 10 rotatable bonds, a drug’s oral bioavailability in rats is lowered, and this is why the rotatable bond count is used as a “drug filter” (Veber et al., 2002). It is still unknown how “rotatable bond filter” is used mechanically because its count does not correspond to the rate of *in vivo* clearance in rats. The filter is justified from an *in vitro* screening prospect, though, as the ligand affinity declines at a rate of 0.5 kcal on average for every two rotatable bonds (Andrews et al., 1984). There are fewer H-bond acceptors, donors, and rotatable bonds in oral medicines. The oral route of administration is favored by these three factors since it is accessible, practical, and convenient (Ahmad et al., 2021). As the present study has established from SwissADME database findings that our synthesized compounds have lesser drug filter (rotatable bonds), fewer H-bond acceptors, and very few H-bond donors. Thus, it is predicted that our synthesized compounds would have better oral bioavailability and therefore, are superior candidates for oral medication.

The compounds with a TPSA of ≥140 A^2^ are poorly absorbed, with fractional absorption being less than 10%, whereas those with a TPSA of 60 A^2^ are highly absorbed, with fractional absorption being greater than 90% (Clark, 1999). Hence, it is further supported by the TPSA values of **C1**-**C10** that all the synthesized compounds could exhibit the best oral absorption.

The consensus Log P_o/w_, also known as the LogP_o/w_ determined by Swiss ADME, is the average of iLOGP, XLOGP3, WLOGP, MLOGP, and SILICOS-IT. The logarithm of the octanol/water partition coefficient is known as Log P_o/w_. The degree of lipophilicity is determined by polarity, molecule size, and hydrogen bonding, and is indicated by a high log P_o/w_ value. These scores are somewhat comparable with log S scores. The range of logP_o/w_ illustrates ideal lipophilicity that is 0<LogP<3 is the optimum range (Bitew et al., 2021). In the present study, the log *p* values of all the synthesized compounds were in the optimum range, thus these findings reflect their partition to lipid compartment except **C4**. In comparison to log S values, our findings predict that all the compounds have moderately to poor solubility in the water compartment except **C4**, which showed insolubility. Furthermore, our research findings have established that **C1** and **C2** can cross BBB which is a good sign for prospects and provides further innovative research guidance for researchers to explore the therapeutic effect of our compounds in the management of neuroinflammation. All the derivatives were found to have high GI absorption. The research findings of our present study have shown that P-glycoprotein is a substrate of all synthesized isoxazole derivatives (**C1**-**C10**). This might connect them to interactions with multiple exogenous or endogenous substances, including medicines. The pharmacological properties of other medications may eventually be influenced by P-glycoprotein interactions (Zhang et al., 2021).

One of the most essential isoforms of the CYP P450 system, CYP3A4, is responsible for the majority of pharmacological and endogenous chemical metabolism. Since all isoxazole derivatives had no impact on CYP3A4, therefore free of said enzymatic interaction. In a similar manner, all the derivatives have no effect on other significant isoforms such as CYP1A2, CYP2C19, CYP2C9, and CYP2D6, with the exception of **C2**, **C5**, **C6**, **C7**, and **C8**, which inhibit CYP2C19 and **C4** and **C9** inhibit CYP 2C9, respectively.

A sufficient plasma concentration is indicated by the bioavailability score. Prior to starting a synthesis or conducting any advanced research, it is necessary to calculate bioavailability and permeability. A pharmaceutical candidate is thus assigned a probability-based score if F>10% in rats (Martin, 2005). All of the derivatives’ bioavailability scores fell within the expected range (0.55). The boiled egg graph demonstrated that there was no problem in the absorption of all derivatives. On the boiled egg graph, the white area denotes intestinal absorption in humans, whereas the yellowish area illustrates CNS penetration. The graph’s grey region indicates non-oral drug absorption if it occurs (Daina et al., 2017).

About 90% of oral medicines which has progressed to phase II (clinical trials) relate to Lipinski’s Rule of Five. The specification of this rule is given by four straightforward physicochemical parameters: log *p* ≤ 5, molecular weight ≤500, H-bond donors ≤5, and H-bond acceptors ≤10 (Lipinski, 2004). Our research findings showed that all the compounds (**C1**–**C10**) comply with numerous generally accepted drug-likeness standards, including Lipinski, Muegge, Ghose, Veber, and Egan, except **C4** and **C7** which showed violations against the Ghose and Egan rules due to molecular weight, MR, and WLOGP (Table 5).

Since none of the synthesized isoxazole derivatives have any PAINS (Pan Assay interference compounds) alerts, they are all eliminated from the list of reactive compounds, frequent hitters, and screen artifacts. During HTS, PAINS have an unchecked behavior of yielding false positive hits. Despite the fact that the mechanism is imperfectly understood, it is connected to protein reactivity and non-covalent interactions (Bolz et al., 2021). Brenk et al. (2008) found 105 fragments that are chemically reactive, poisonous, metabolically unstable, or likely to have poor pharmacokinetics, and Swiss ADME produced a structural alert for those fragments. With this, a problematic fragment in a particular molecule can be detected. All the compounds were found to have no Brenk alert, thus all are excluded from the list of 105 fragments that are chemically reactive, poisonous, metabolically unstable, or likely to have poor pharmacokinetics.

The capability of a compound to act as “lead” during the drug discovery process is represented by the lead likeness parameter. **C1** has lead likeness capability as its MW ˂ 350. All the remaining compounds **C2**-**C10** disobeyed one of the lead likeness rules as their MW˃350 except **C4** which violated 2 rules of lead likeness such as MW > 350, XLOGP3>3.5. To get superior pharmacological outcomes, their pharmacophores can be further changed based on SAR. Isoxazole derivatives (**C1**-**C10**) have been synthesized in the laboratory. The SwissADME database’s forecast of its synthetic accessibility. Isoxazole derivatives were given scores in the range of 3.99–4.74 by the Swiss ADME database, indicating simple step processes for synthesis. Those compounds with a score of 10 require challenging synthesis protocols (Ahmad et al., 2021).

## 5 Conclusion

In the current research, a diverse array of ten isoxazole derivatives (**C1**-**C10**) were designed and synthesized by Claisen Schmidt condensation reaction. In the present research, *in vitro* anti-inflammatory activities, i.e., both COX-1 and COX-2 enzymes inhibition assays established that all the tested compounds showed poor selectivity towards COX-1 enzyme whereas the tested compounds exhibited a strong effect against COX-2 enzymes anti-inflammatory assay and were found a good candidate for anti-inflammatory effect. The binding orientations and binding energy results also showed the selectivity of compounds towards COX-2. *In silico* molecular docking studies coupled with molecular dynamic simulations were also performed to rationalize the time-evolved mode of interaction of selected inhibitors inside the active pockets of target COX-2. Our findings confirmed that almost all the synthesized compounds have high GI absorption. Lipinski’s rule of five was satisfied by all the ten synthesized isoxazole derivatives. This indicates that their molecular structure makes them suitable for oral medications. It was further concluded that the pharmacokinetic profile of the compounds was improved. The research findings have also shown that P-glycoprotein is a substrate of all synthesized isoxazole derivatives (**C1**-**C10**). The results of this research should be extended to the development of innovative and efficient isoxazole derivatives in the future using SAR-based drug design techniques. Moreover, **C1** and **C2** were found to have improved GI absorption and BBB permeability, which should be helpful for future drug discovery research. Furthermore, from the result finding and the above discussion it was concluded that among the tested compounds, **C6**, **C5**, and **C3** were found most potent and showed excellent *in vitro* COX-2 anti-inflammatory effects and were found excellent selective COX-2 inhibitors which will be further investigated via *in vivo* anti-inflammatory activities. Moreover, to further support our findings of the computational research and *in vitro* studies, an *in-vivo* pharmacokinetic profile is suggested in the future.

## Data Availability

The raw data supporting the conclusion of this article will be made available by the authors, without undue reservation.

## Abbreviations

ADME, Absorption, distribution, metabolism and excretion; BBB, Blood-brain barrier; CYP, Cytochrome; COX-2, Cyclooxygenase-2; FLEX, Flexibility; F. Csp^3^, No. of sp^3^ hybridized carbon out of total carbon counts; GI, Gastrointestinal; INSATU, Insaturation; INSOLU, Insolubility; LIPO, Lipophilicity; Log P_o/w_, log of the octanol/water partition coefficient; MW, Molecular weight; MR, Molar refractivity; NMR, Nuclear magnetic resonance; nHA, No. heavy atom; nAHA, No. aromatic heavy atom; nRB, No. of rotatable bonds; nHBA, No. H-bond acceptors; nHBD, No. H-bond donors; PAIN, Pan assay interference compounds; PK, Pharmacokinetics; POLAR, Polarity; TPSA, Topological Polar Surface Area.
